# Research on the Optimal Regulation of Sudden Water Pollution

**DOI:** 10.3390/toxics11020149

**Published:** 2023-02-03

**Authors:** Honglei Ren, Fei Lin, Yuezan Tao, Ting Wei, Bo Kang, Yucheng Li, Xian Li

**Affiliations:** 1 College of Civil Engineering, Hefei University of Technology, Hefei 230009, China; 2 School of Resources and Environmental Engineering, Anhui University, Hefei 230601, China; 3 Institute of Intelligent Machines, Chinese Academy of Sciences, Hefei 230031, China; 4 School of Resources and Environmental Engineering, Hefei University of Technology, Hefei 230009, China

**Keywords:** sudden water pollution, emergency regulation, parameter quantification method, NSGA-II, optimized partition, water supplying time

## Abstract

For the needs of the whole region’s emergency regulation of the nullah sudden water pollution event, the emergency regulation strategy of the accident section and upstream and downstream of the sudden water pollution event is studied. For the accident section, the duration of the whole emergency event is calculated using the parameter quantification method; for the upstream of the accident section, the NSGA-II is used to adjust the gate opening to ensure the water level stability of the upstream pools; for the downstream section, the optimized partition method is used to identify the unfavorable pools and close the unfavorable pool to extend the water supply time. Based on the example of an emergency event in the section of the Liyanghe gate–Guyunhe gate of the middle line project, the research results are as follows: the accident section is identified as the Xiaohe gate–Hutuohe gate, the upstream of the accident section is the Liyanghe gate–Xiaohe gate, and the downstream of the accident section is the Hutuohe gate–Gangtou Tunnel gate. The duration of the emergency event in the accident section is 7.9 h; the maximum average water level deviation before the gate upstream of the accident section is 0.05 m; two unfavorable canal pools are identified in the stream of the accident section, and the water supply time of the unfavorable pools is extended by 6.13 and 5.61 d.

## 1. Introduction

Open channel water transfer is one of the most commonly used water transfer methods in large-scale water transfer projects. Natural channels or artificial channels are used as the main water transfer carriers, which are favored by many water transfer projects at home and abroad because of their characteristics of small investment, low operation costs and large water transfer flow [1]. In open channel water transfer projects, water quality safety is an important guarantee for water transfer projects to exert economic and social benefits. Sudden water pollution events can currently be divided into two categories; the first is the event of a large number of pollutants entering the water body caused by man, and the second is the sudden change in climate leading to a sudden deterioration of water quality. Among them, many scholars have studied the impact of sudden climate change on water quality. Bastincich et al. [2] analyzed the availability and impact of climate change on surface water; Lasagna et al. [3] analyzed the impact of an arid climate on surface water; Chen et al., Lu et al. and Fei et al. [4,5,6] analyzed the way climate change affects water quality and proposed corresponding management countermeasures; and Maurizio et al. [7] analyzed the impact of climate change on groundwater quality.

In recent years, sudden water pollution events have occurred frequently, which not only bring immeasurable impacts on the environment but also threaten social and economic development, causing people’s concerns about water safety in open channel water transfer projects [8]. A sudden water pollution accident refers to an accident that occurs suddenly and causes a large number of pollutants to enter the water body due to man-made or natural factors [9], which can lead to the deterioration of water quality, affect the safety and production of water in daily life, cause huge economic losses, harm the ecological environment and bring bad social influences [10].

In recent years, the frequent occurrence of sudden water pollution events not only brings immeasurable impacts on the environment but also threatens social and economic development, causing people to worry about the water safety of the nullah water transfer project. Some scholars have conducted a series of studies on this issue [11,12,13]. Tang et al. [14] studied the pollutant transport and diffusion rules under normal water transfer conditions of the middle route of the South-to-North Water Transfer Project. Huang Huiyong et al. [15] proposed that the emergency dispatching scheme in the face of sudden water pollution accidents should follow the principle of “the right of two evils, the lesser one”. It is possible to temporarily close the downstream control gate and open the discharge gate for emergency water discharge without considering the operation safety of the canal pool. Lian et al. [16] analyzed the flow movement state and pollutant diffusion law in the channel section under different gate closure regulation methods and gate closure event conditions and proposed that synchronous gate closure should be adopted under sudden water pollution events, and the gate closure time should be more than twice the flow propagation time in the channel section. Fang et al. [17] simulated the emergency treatment mode under prominent water pollution events for the first section of the middle route of the South-to-North Water Diversion Project, including two ways: not opening the discharge gate and closing the control gate to control the pollution mass in the accident section for subsequent treatment and using the discharge gate to discharge the polluted water from the canal pool. For the middle route of the South-to-North Water Diversion Project, Nie [18] proposed a method to increase the target water level of the control gate upstream of the accident section as much as possible to save the excess water in the upstream channel section of the accident section in the channel pool. Li et al. [19] took the Huaidian gate in Yinghe River as the research object, constructed a two-dimensional hydrodynamic and water quality model of the coupled gate control system and studied and analyzed the pollutant diffusion law under the control gate regulation.

Due to the sudden water pollution event accident section, the accident section upstream and downstream emergency regulation and control objectives are different, so for the sudden water pollution event region-wide emergency regulation and control is more difficult. The accident section of the sudden water pollution control goal is to retreat as quickly as possible and through the operation of the gate, so that the impact of pollutants on other canal pools is reduced as far as possible. The accident section of the upstream control goal in the flow-switching process of water level stability, when there is a sudden water pollution event, is bound to reduce the flow; in this process, if the gate adjustment is not appropriate, it is very easy to cause fluctuations in water levels and damage to the buildings of the canal pool. The downstream regulation of the accident section aims to increase the water supply time of the downstream canal pool as much as possible because when the accident section occurs, in order to prevent the leakage of pollutants from the accident section, it is bound to close the gates downstream of the accident section; when the incoming water downstream of the accident section is suspended, it is bound to affect the safety of the water supply downstream.

Due to the different and complex regulation objectives of the accident section of the sudden water pollution event and the upstream and downstream, there are few studies for the emergency regulation of the accident section and the upstream and downstream [20]. Some scholars [21] have already achieved some results when facing sudden water pollution events, but the existing results are mainly for the emergency regulation of the accident section and the upstream of the accident section. In order to form a complete set of emergency regulations and control strategies for sudden water pollution, this paper studies the emergency regulations and control methods for the accident section, upstream and downstream of the accident section for sudden water pollution events in the middle line of the South–North Water Transfer Project. When the accident section has a sudden water pollution event, as the middle line project is a gate group tandem system, if the gate closing time downstream of the accident section is greater than the time of pollutant diffusion, it will lead to the transfer of pollutants to the next canal pool. Therefore, understanding the diffusion law and diffusion range of pollutants can provide information support for the regulation and control of upstream and downstream gates in the accident section. Therefore, this paper proposes three methods of pollutant dispersion quantification, a gate optimization control method and optimal zoning to realize emergency regulation and control of the accident section, upstream and downstream of the accident section, respectively, for unexpected water pollution events.

This paper will study the emergency regulation and control of sudden water pollution from three aspects. Firstly, we will give an overview of the study area and the research methods of different accident sections, then we will analyze the use cases of different accident sections, and finally, we will extract conclusions from the case studies for discussion.

## 2. Materials and Methods

### 2.1. Study Area Overview

**Figure 1:**
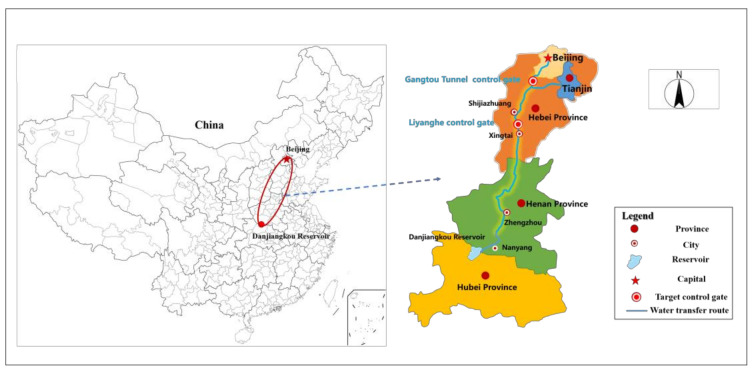
Schematic map of the location of the study area.

The middle route of the South-to-North Water Diversion Project diverts water from Danjiangkou Reservoir in the middle and upper reaches of the Han River to the north, passing through Henan and Hebei provinces, connecting Haihe River, Yellow River, Huaihe River and Yangtze River basins [22], and transporting “southern water” to Tuancheng Lake in Beijing and the Outer Ring River in Tianjin [23]. The project is 1432 km long, and 64 bleeder gates, 97 bleeders, a pump station and numerous inverted siphons, aqueducts and pressure-free culverts are positioned along the route [24]. As the South–North Water Transfer Project spans three provinces and two cities, the climatic conditions vary significantly. In the area north of Yellow River, there is an ice period due to the low temperature in winter, while south of Yellow River, there is no ice period. However, the midline project belongs to the monsoon climate, with high temperatures and rain in summer and low temperatures and less rain in winter. Due to high temperatures and rainfall in summer, the runoff effect is obvious, so summer is also the key time period to prevent sudden water pollution. It has been 8 years since the middle route project was officially opened on 12 December 2014. Water has been transported safely for more than 2000 days, and more than 50 billion cubic meters of water have been transported to the north, benefiting 85 million people along the route [25]. The area selected for this study is the Liyanghe control gate–Gangtou Tunnel control gate section of the South–North Water Transfer Central Project, with 12 control gates, 18 water diversions and 12 water withdrawals. The study area is shown in Figure 1.

### 2.2. Research Method

In case of sudden water pollution events in the accident section, the downstream gate of the accident section is bound to be closed to prevent pollutants from leaking out. However, because the middle line project is a series system of gate groups, if the closing time of the downstream gate in the accident section is longer than the time of pollutant diffusion, pollutants will be transferred to the next channel pool, resulting in pollutant leakage. Therefore, understanding the diffusion law and diffusion range of pollutants can provide information support for the regulation of downstream control gates.

In this section, the pollutant diffusion quantification method is proposed to accurately describe the pollutant diffusion process and scope of the sudden water pollution, and the accident section and downstream of the accident section are divided according to the diffusion scope. After the division is completed, unfavorable channels and pools are identified using the method of constant flow model and optimized zoning, and water supply zones are divided to ensure the safety of water supply downstream of the accident section and complete the emergency control downstream of the accident section.

#### 2.2.1. Accident Section Regulation Method

After pollutants enter the water body, they generally need to go through three stages [26,27,28], namely, the core area of the jet flow, the diffusion area and the discrete area. In this paper, we consider the pollutant distribution uniformity and do not consider the degradation function (namely, discrete zone), select pollutants’ longitudinal length of the peak, peak pollutant transport distance and pollutant concentration as the characteristic parameters of sudden water pollution by calculating longitudinal length of contaminants and pollutants peak transport distance so as to determine the extent of contamination.

Pollutant concentration can be expressed as follows [29]:

(1) $$C\left(x,t\right)=C_{0}\frac{v}{\sqrt{4\pi D_{L}t}}\exp\left(\frac{{\left(x-vt\right)}^{2}}{4D_{L}t}\right)$$

where *C*(*x*,*t*) is the pollutant concentration along *x* at time *t*, mg/L; *C*_0_ is at *x* = 0, and the instantaneous concentration of pollution source is mg/L; *C*_0_ = *M*/*Q*; *M* is the total amount of pollutants released instantaneously, g; *Q* is discharge, m^3^/s; *A* is river sectional area, m^2^; *D_L_* is dispersion coefficient, m^2^/s; and *x* is the distance between the feed and the drop point, m.

According to Equation (1), pollutant concentration is normally distributed. According to the characteristics of normal distribution, pollutant concentration takes the maximum value. For the channel with low flow velocity, the distance of pollutant concentration transport is

(2) D=60vT

where *D* is pollutant concentration transport distance, m; *v* is the average velocity of the section, m/s; and *T* is travel time, s.

Pollutant concentration is also normally distributed. According to the characteristics of normal distribution, the diffusion width is defined as (m is constant). The idea of determining the dispersion coefficient according to the known tracer emission is as follows:


(3) $$D_{L}=\frac{1}{2}\frac{\partial \sigma^{2}}{\partial t}$$

By integrating Equation (3), the longitudinal stretching velocity of pollutants can be obtained:

(4) $$v=\frac{m\sigma }{t}=a\sqrt{2}D_{L}{}^{0.5}t^{-0.5}$$

where *a* = *m*/*s*.

The vertical distance of pollutant diffusion can be obtained by integrating Equation (4):


(5) $$W=\int_{o}^{T}vdt=2a\sqrt{2}D_{L}{}^{0.5}T^{0.5}$$

According to the quantification process of Formula (5) by Yan Long [30], it can be known that:

(6) $$W=\left[12+\ln\left(\frac{M}{10}\right)\right]\sqrt{2D_{L}}T^{0.455}$$

(7) $$D_{L}=m\times 0.011\frac{v^{2}B^{2}}{h\sqrt{ghJ}}$$

where *W* represents the distance of pollutant diffusion, m; *M* and *D_L_* have the same meaning as above; m usually takes 1; *B* and *h* represent the average width and average water depth of the channel, respectively, m; *J* stands for hydraulic slope; *v* represents the average velocity of the section, *v* = *Q*/*A*, m/s; *T* represents the time for the pollutant to disperse, s; *g* represents gravitational acceleration, m/s^2^.

The distribution characteristics of pollutant concentration can be seen from the above, and the distance between pollutant front and pollution source can be calculated using Formulas (2) and (6):

(8) $$X=D+\frac{W}{2}$$

where *X* represents the distance between the pollutant front and source of pollution, m; and *D* and *W* have the same meaning as above.

After completing the analysis of pollutant diffusion, it is necessary to use the water outlet to quickly discharge the pollutant from the canal pool to ensure the safety of water quality. In actual projects, it takes a certain amount of time to open the water back outlet, and it is difficult to summarize the opening time of the water back outlet in view of the difference in the size of the water back outlet. Therefore, this paper adopts the method proposed by Jiankui Liang [31] to open the water back outlet 500 m before the pollutant front reaches the water back outlet. The default diffusion form of pollutants is uniform diffusion, so the volume of pollutants in the diffusion process is:

(9) V=B×h×W

where *V* represents the volume of water contaminated by the contaminant in the process of diffusion, m^3^; *B* represents the width of channel, m; and *h* represents the depth of channel, m.

After the volume of pollutant diffusion is calculated, the water is discharged through the discharge port (default is the design flow rate), so that the water discharge time of the whole pollution event can be calculated. The formula for calculating the draining time is as follows:

(10) t=V/q

where *t* represents duration of dewatering, s; and *q* represents design flow rate of the drain, m^3^/s.

The study area is divided into 11 channels and pools by 12 control gates, and the water surface line of each channel pool is affected by the designed water depth and current flow, as well as by the coupling between adjacent channels and pools [32]. When calculating the storage capacity of the canal pool, the water surface line of each canal pool is considered to be constant, and the constant flow of each canal pool is calculated.

The basic differential equation of constant gradient flow is as follows:

(11) $$\frac{dE_{s}}{ds}=i-J$$

where *E_s_* = *h*cos*θ* + (*αv*^2^)/(2*g*) represents potential energy of a section; *h* represents the depth of channel, m; *θ* represents angle between channel and horizontal line; *v*, *g* and *J* have the same meaning as above; *i* represents degree of slope at the bottom of the canal, *C* represents Chezy coefficient, m^1/2^/s; and *R* represents hydraulic radius, m.

The integration of Equation (11) is as follows:


(12) $$E_{sd}-E_{su}=\int_{0}^{1}\left(i-J\right)ds=\Delta s\cdot \left(i-\bar{J}\right)$$

Equation (12) can be expressed in the form of finite difference:


(13) $$E_{sd}=E_{su}+\Delta s\left(i-\bar{J}\right)$$

In the formula, *E_sd_* represents wanting to find the cross-sectional energy of the solution; *E_su_* represents knowing the specific energy of the cross section; Δ*s* represents the distance between two sections, m; and $\bar{J}$ is the average hydraulic slope between two sections.

Taking the known section as the control section, the desired section can be deduced according to Formula (13). The water surface line can be determined using continuous deduction.

#### 2.2.2. Accident Section Upstream Regulation Method

In the event of a sudden water pollution accident, in order to reduce the impact of incoming flow on the spread of pollutants in the accident section and reduce water disposal, the control gates upstream of the accident section will choose to suppress or interrupt the flow, but the upstream flow is still very large, so all the upstream pools will have “excess flow”. The middle route of the South-to-North Water Diversion Project uses the normal water level control method before the gate; when the downstream flow is reduced, the water surface line is different from the demand, resulting in “excess storage capacity”. In the face of the ensuing threat of overflow, it is necessary to reduce or cut off the incoming flow from the upstream the first time and combine with the receding gate to recede to ensure the water level of the canal pond is stable and ensure the safety of water transmission.

Due to the unequal relationship between the upstream flow and the outflow, there is “excess storage” in the canal pond. In order to ensure the stability of the water level in the canal pond, it is necessary to use the canal pond’s receding mouth to remove the excess water from the channel. Therefore, in this paper, while considering the water level stability in front of the channel gates, the number of gate adjustments and the amount of water returned are coupled into the model as objective functions to establish a multi-objective optimal scheduling model for sudden water pollution events.

In this paper, the NSGA-II optimization algorithm is used to optimize the amount of receding water as well as the number of adjustments, and the objectives of optimization are as follows:

(1) Minimum average deviation of operating water level and target water level

(14) $$e_{\min}=\frac{\sum_{t=1}^{T}\left|Z_{gt}-Z_{ot}\right|}{T}$$

where *e*_min_ indicates the average deviation of the operating water level and the target water level, m; *T* indicates the optimization time, h; and *Z_gt_* and *Z_ot_* indicate the target water level and the measured water level, respectively.

(2) Minimal water withdrawal

(15) $$\min T_{w}=\min\left\{\frac{\sum_{t=1}^{T}Q_{t}}{T}\right\}$$

where *T_w_* is a measure of the amount of water receding, and *Q_t_* indicates the amount of water receding, m^3^/s.

As the optimization is the regulation process of the upstream throttle gate, the amount of overflow variation of the gate is selected as the decision variable according to the needs of the actual engineering dispatch. The constraints of the optimization model are selected as water level constraint and flow rate constraint.

(1) Water level constraint
  (16) $$Z_{\min}\leq Z_{ot}\leq Z_{\max}$$
  where *Z*_min_ and *Z*_max_ indicate the minimum and high operating water level of the canal pool, m.
(2) Hourly water level variation
  (17) $$Z_{ot+1}-Z_{ot}\leq Z_{h}$$
  where *Z_h_* indicates the hourly maximum water level variation, often taken as 0.15 m.
(3) Flow constraints
  (18) $$Q_{\min}\leq Q\leq Q_{\max}$$
  where *Q*_min_ and *Q*_max_ indicate the minimum overflow and maximum overflow of the throttle gate, m^3^/s.

#### 2.2.3. Accident Section Downstream Regulation Method

**Figure 2:**
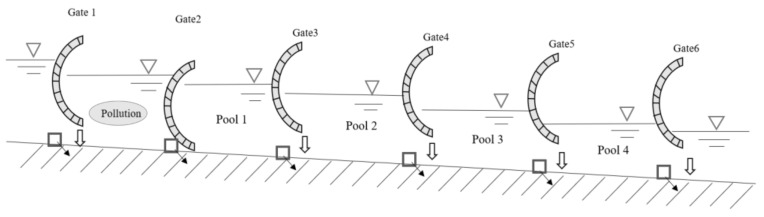
Local partition method.

The normal water transfer status of the open channel project usually adopts the method of local zoning (Figure 2) to supply water to the downstream city. The method of local zoning means that the upstream control gate of the current canal pond and the downstream control gate near the first water diversion are used as a water supply zoning, and the water of the current canal pond is only supplied to the current canal pond or the downstream water diversion near it.

Major cities along the open channel water transfer project have large water demands, and the corresponding water distribution flow is larger. When local bleeder water supply method is used, the key bleeder has a short water supply time compared with other bleeder ponds. This paper proposes an optimized partition method to extend the water supply time of the key bleeder ponds. This partition method mainly reflects the water distribution of the canal pool through the water supply time of the canal pool. When the water distribution time of the accumulated canal pool is the minimum value, it means that the water distribution volume of the last accumulated canal pool is large, and this canal pool is called the unfavorable canal pool. This adverse canal pool, because of the large amount of water, cannot continue to supply water to downstream; if it continues to the downstream water supply, it will aggravate the drainage situation of the canal pool, resulting in less drainage time, so closing the downstream gate will maximize the adverse canal pool water supply time. In general, the unfavorable canal pool and the important canal pool can be regarded as the same.

**Figure 3:**
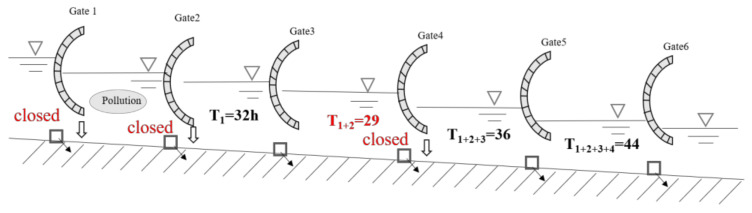
Optimized partition method.

Optimal partition water supply method in canal pool is shown in Figure 3. Firstly, the hydrodynamic model is used to calculate the storage capacity of each channel pool in the study section, and Formula (17) is used to calculate the water supply time after the current channel pool is successively accumulated downstream. Then, Formula (18) is used to find out the unfavorable canal pool. Then, close the drainage pool downstream of the gate, adverse bad drainage pool and, above all, the drainage pool as a partition of water supply; the drainage pool, under the circumstances of a drainage basin, will be the new first drainage pool. Repeat the above work to identify adverse drainage pools until the last ditch drainage pool, and this shows the optimal partition work is finished.

(19) $$\begin{array}{l} T_{1}=\frac{V_{1}}{Q_{1}}\\ T_{1+2}=\frac{V_{1}+V_{2}}{Q_{1}+Q_{2}}\\ \cdots \cdots \\ T_{1+2+\ldots +N}=\frac{\sum_{i=1}^{N}V_{i}}{\sum_{Q=1}^{N}Q_{i}} \end{array}$$

where *T*_1_ represents water supply time of the first canal pool, s; *T*_1+2_ represents water supply time after the accumulation of the first and second canal pools, s; *T*_1+2+...+*N*_ represents the water supply time after the first channel pool is successively added to the Nth channel pool, s; *V*_1_ represents that the first canal has storage capacity, m^3^; and *Q*_1_ represents the first channel distributes water to the pool, m^3^/s.

(20) $$T_{1+2+\ldots m}=\min\left\{T_{1},T_{1+2},\ldots ,T_{1+2+\ldots m},\ldots ,T_{1+2+\ldots m+\ldots +N}\right\}$$

where *m* represents the m-th canal pool as the unfavorable canal pool.

Assuming that *N* identifies bad drainage pool, then adopt the method of optimal partition; it will be divided into *N* + 1 partition. With the *N* bad drainage pool, adopt the optimum partition method, and partial partition is against the same drainage basin water supply time comparison, thus providing comparative results for optimized partition and local partition of the same bad drainage pool water supply time.

## 3. Results

For sudden water pollution events of the canal pool, the polluted canal pool and the unpolluted canal pool are divided into the accident section, the upstream of the accident section and the downstream of the accident section. In the accident section, the control strategy of the control gate and the discharge outlet is determined by analyzing the diffusion distance of pollutants, so that the pollutants can be discharged from the water transmission channel in the fastest time. Through the flow control method upstream of the accident section, the operation safety of the upstream canal pool of the accident section is ensured during the accident period. The downstream of the accident section is divided into water supply units based on the optimized zoning method to extend the water supply time of the adverse canal pool.

In this paper, the Liyanghe control gate (pile No. 868+402)–Gangtou Tunnel control gate (pile No. 1112+139) is selected as the research channel section. There are 11 canal pools composed of 12 control gates, and the Liyanghe control gate and Wuhe control gate are taken as Pool 1. The location of the water pollution incident is at the Xiaohe control gate–Guyunhe control gate (Pool 4, pile No. 953+515). The total amount of pollutants was 1 t, the base width was 22.5 m, the slope coefficient was 2.5, the design depth was 6 m, the slope degree of the canal bottom was 0.00005, and the roughness ratio was 0.015. The dispersion coefficient is estimated at 3.43 m^2^/s, and the flow conditions are selected from real-time monitoring information, and the water separation rate from the channel bleeder is obtained according to the real-time monitoring information.

### 3.1. Emergency Control of Accident Section

In the daily dispatching process of the middle route of the South-to-North Water Diversion Project, the closing time of the gate is closely related to the operation safety of the canal pool. However, in emergency dispatching, the gate will be closed at the speed of 0.4 m/min without considering the water level constraints of 0.15 m/h and 0.3 m/d, so the specific closing time is closely related to the real-time opening of the gate. The opening degree of the gate is adapted to the selected flow condition. The specific information is shown in Table 1.

Table 1 shows that through the gate of the original opening and the closing of the gate under the emergency state, we can calculate that the closed pool downstream of gate 1 time is about 5 min and then calculating the gate closing time into the Formulas (2), (6) and (8), it can be calculated from the gate fully closed time that the peak concentration of the pollutant diffusion distance is about 0.3 km. The longitudinal distance of pollutant diffusion is about 0.825 km, and the distance of pollutant front diffusion is about 1.13 km, which is far less than the distance between the pollutant source and the downstream control gate. However, Pool 1 has no discharge outlet, so the accident section is Pool 4 and Pool 5 combined with the two aspects. One should close the downstream control gate of Pool 5 (the Hutuohe control gate) and ensure that the downstream control gate of Pool 4 (the Guyunhe control gate) is fully opened. The process of accident segmentation is shown in Table 2.

As the downstream control gate of Pool 4 is fully opened, Pool 4 and Pool 5 form a new channel pool. Based on the pile number, it is calculated that the distance between the source of pollution and the outlet of the Hutuohe is 24.29 km before the 500 m. Equation (9) is used to calculate that the water volume in the process of pollutant diffusion is about 2426 × 103 m^3^. It is calculated that the time for all pollutants to exit the canal pool is about 7.9 h. Accordingly, the emergency control of the accident section is completed. The regulation process of the accident section is shown in Table 3.

### 3.2. Emergency Control Upstream of the Accident Section

As the location of the sudden water pollution is located in the section of the Xiaohe control gate–Guyunhe control gate, the natural Xiaohe control gate is chosen to be closed, then the upstream of the accident section is the Liyanghe control gate–Wuhe control gate (Pool 1), the Wuhe control gate–Huaihe River (I) control gate (Pool 2), and the Huaihe (I) control gate–Xiaohe control gate (Pool 3), and the specific information of the drainage basin is shown in Table 4.

**Figure 4:**
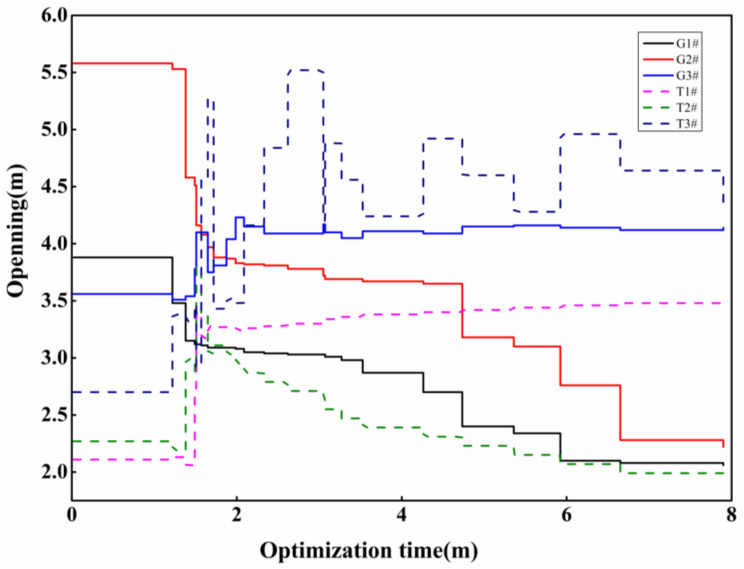
Variation of the opening degree of sectional gates and backwater gates. (The solid line is the control gate, and the dotted line is the backwater gate).

**Figure 5:**
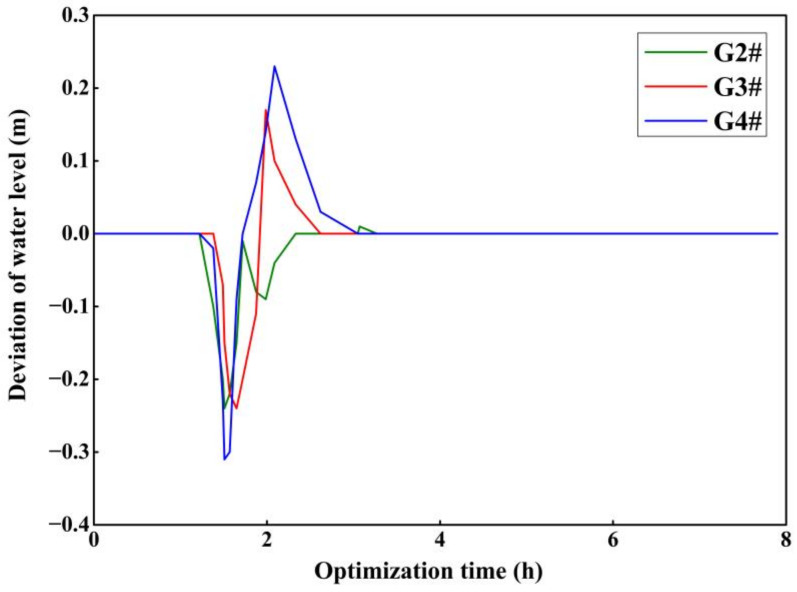
Water level change before the gate.

There are three gates upstream of the accident section, and the optimization period is 7.9 h for the receding time of the accident section, so there are 24 optimization variables set. The optimization algorithm is used to optimize the opening degree of the three upstream gates and the corresponding backwater outlet in the drainage basin, and the change in the water level in front of the gates in the drainage basin is shown in Figure 4 and Figure 5.

From Figure 5, it can be seen that the optimal solution of the model is obtained through the multi-objective model calculation, and the model ensures that the pre-gate water level of all the canals and ponds upstream of the accident section is stable around the design water level by frequently regulating the gates. For the second restraint gate upstream of the accident section, the maximum deviation of the water level reached 0.24 m, and the average deviation was only 0.05 m; for the third restraint gate, the maximum deviation of the water level was 0.24 m, and the average deviation of the water level was 0.03 m; and for the fourth restraint gate, the maximum deviation of water level was 0.3 m, and the average deviation of the water level was 0.01 m. This proves that the multi-objective optimization algorithm can quickly achieve the optimal regulation of the upstream of the accident section.

### 3.3. Downstream Emergency Regulation of Accident Section

In case of sudden water pollution in the accident section, the last control gate of the accident section will be closed, and the downstream of the accident section will supply water continuously with its own storage capacity. If an accident period of extreme water pollution occurs, the gate closing time is longer, and the accident section downstream points with a large amount of water to the drainage pool. Water supply security is difficult to guarantee and is based on the optimal partition method: identify the accident section downstream to all bad drainage pools and ensure the bad drainage pool gate is closed and that the adverse impact on downstream water dropping into the drainage pool is kept to a minimum, whilst extending the downstream drainage pool water supply time.

For downstream of the accident section, first, the constant flow model is used to calculate the storage capacity of all the channel pools downstream of the accident section. The specific storage capacity of the channel pools is shown in Table 5.

According to the information in Table 5, and by using Equations (14) and (15), the unfavorable channel pool downstream of the accident section can be calculated, and the unfavorable channel pool water supply time under the optimized zoning method can be calculated. At the same time, according to the method of local partition, the water supply time of each channel pool is calculated, and the water supply time of the two methods is compared for the unfavorable channel pool.

**Figure 6:**
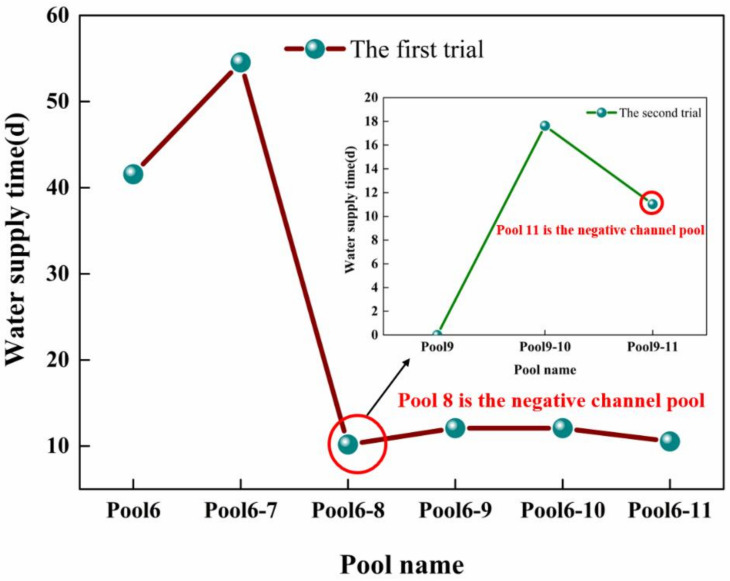
The result of the trial calculation.

According to the trial calculation results in Table 6 and Table 7 and Figure 6, the unfavorable canal pools downstream of the accident section are the Shahe (north) control gate–Tanghe control gate and the Puyanghe control gate–Gangtou Tunnel control gate, respectively. So in accordance with the optimization of the zoning method, this can be divided into the lower reaches of the Hutuohe control gate–Tanghe control gate and the Tanghe control gate–Gangtou Tunnel control gate as two large canal pools. The water supply time of each canal pool downstream of the accident section was calculated according to the method of local partition.

**Figure 7:**
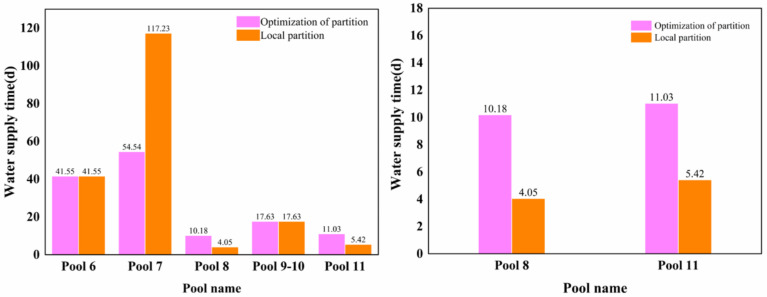
Water supply time of the two zoning methods and water supply time of the most unfavorable channel pool.

As can be seen from Table 8, according to the local partition method, the water supply time of each channel pool is compared between the two methods in the unfavorable channel pool. It can be found that the Shahe (north) control gate–Tanghe control gate is identified as the unfavorable channel pool using the optimized partition water supply method, and its water supply time is 10.18 days. However, the water supply time of the lower reaches of the Shahe (North) control gate–Tanghe control gate was 4.05 days, which extended the time of the canal pool by about 6.13 days. The second unfavorable canal pool was identified as the Puyanghe control gate–Gangtou Tunnel control gate with a water supply time of 11.03 d using the method of optimized zoning, but the water supply time of the Puyanghe control gate–Gangtou Tunnel control gate was calculated to be 5.42 d using the method of local zoning. Compared with the method of local zoning, the water supply time of the canal pool was prolonged by about 5.61 d using the optimized zoning method. The calculation results are shown in Figure 7.

## 4. Conclusions

The multi-objective optimization algorithm (NSGA-II), the quantification method of pollutant characteristic parameters and optimization partitioning are three methods which can meet the emergency regulation and control needs of different objectives in the whole region of a sudden water pollution event. This paper takes the sudden water pollution event in the Liyanghe–Gangtou Tunnel section of the South–North Water Transfer Central Canal as an example and analyzes the emergency regulation and control effects of the three methods in the accident section, upstream of the accident section, and downstream the accident section, respectively.

For the accident, the characteristic value was proposed based on a pollutant quantitative method, through the pollutants’ concentration, transport distance bandwidth, diffusion forward and diffusion distance. Three characteristic values of the diffusion of the pollutants from the generalized process generate the accident period of the gate control strategy, thus rapidly dividing the accident and accident downstream, realizing fast regulation of sudden water pollution accidents.

For the upstream of the accident section, the multi-objective genetic algorithm was used to optimize the receding water volume and water level in the upstream of the accident section. By optimizing the opening degrees of the three gates and the receding inlets in the upstream, the average deviation of the water level in the three canal ponds in the upstream is 0.05, 0.03 and 0.01 m, respectively, which effectively ensures the operational safety in the upstream of the accident section.

We sectioned downstream of the accident and put forward the optimal partition method. Through the period of time of the trial, the downstream water supply during the accident identified the adverse drainage pool (the Shahe (north) control gate–Tanghe control gate and the Puyanghe control gate–Gangtou Tunnel control gate), and we used the local partition method to calculate the bad drainage pool water time and compared the two methods of supply. It was found that optimized zoning could prolong the water supply time of the unfavorable canal pool by 6.13 and 5.61 d, respectively.

There are differences in the regulatory objectives of different regions of sudden water pollution, which leads to the fact that the same methods cannot be used in different regions facing sudden water pollution events, so the three methods proposed in this paper are oriented to different regulatory objectives. Thus, the three methods mentioned in this paper can also be applied individually to a certain application scenario. For example, the South–North Water Transfer Project is a water transmission channel, so flow switching is more common due to the change in water consumption downstream, but the water level control of the canal pool is a difficult problem; however, the multi-objective genetic algorithm proposed in this paper can achieve smaller fluctuations under flow switching as long as the regulation target is converted.

## Figures and Tables

**Table 1:** Study the basic parameters of canal pool.

Pool Number	Length of Pool	Bottom Width	Opening	Channel Flow Velocity	Depth of Water	The Downstream Flow	Water Flow
Pool 4	23.78	22.5	2.02	1.03	4.5	141.21	16.21
Pool 5	9.73	22.2	1.57	1.26	4.5	121.32	0
Pool 6	12.05	20.4	2.34	1.02	4.21	121.32	0.82
Pool 7	15.19	20.4	2.20	1.02	4.19	120.50	0.17
Pool 8	19.54	20.6	2.13	0.86	4.19	120.33	7.16
Pool 9	9.23	18.9	2.57	0.85	4.21	113.17	0
Pool 10	25.72	18.7	2.78	0.97	4.17	113.17	2.79
Pool 11	13.18	23.0	1.77	1.32	3.9	110.38	6.28

**Table 2:** Calculation process of accident section determination.

The Accident Section	Gate Closing Time (s)	Channel Flow Velocity (m/s)	Peak Concentration Diffusion Distance (km)	The Longitudinal Distance of Diffusion (km)	Forward Diffusion Distance (km)	Distance between Source Front and Downstream Control Gate (km)
Pool 4	300	1.03	0.3	0.83	1.13	16.86

**Table 3:** Control results of accident section.

Name of Gate	Pollution Source Pile Number	Pile Number of Drain Port	Pile Number 500 m Before Water Retreat	Designed Discharge Rate of the Drain (m^3^/s)	Volume of Diffused Water (m^3^)	Control Gate Control Strategy	Time (h)
Xiaohe (946+602)	953+515	977+801	977+301	85	2426 ∗ 103	no change	7.9
Guyunhe (970+379)						Fully open	
Hutuohe (980+116)						Closed	

**Table 4:** Basic parameters of the drainage basin upstream of the accident sectio.

Channel	Length of Channel (km)	Flow (m^3^/s)	Max of Flow (m^3^/s)	Water Level (m)	Target Water Level (m)	Max Flow Rate of Backwater Gate (m^3^/s)
Pool 1	30.84	147.26	240	82.36	82.66	110
Pool 2	21.45	144.72	240	80.73	81.03	120
Pool 3	25.91	141.72	240	79.21	79.51	110

**Table 5:** Volume of downstream canal pool in accident section.

Pool Number	The Name of Channel	Volume (10^3^ Km^3^)	Water Flow (m^3^/s)
Pool 6	Hutuohe–Cihe	294.34	0.82
Pool 7	Cihe–Shahe (north)	172.19	0.17
Pool 8	Shahe (north)–Tanghe	250.50	7.16
Pool 9	Tanghe–Fangshuihe	133.58	0
Pool 10	Fangshuihe–Puyanghe	291.33	2.79
Pool 11	Puyanghe–Gangtou Tunnel	153.51	3.28

**Table 6:** Results of the first trial calculation.

Pool Number	The Name of Channel	The Sum of Volume (10^3^ Km^3^)	The Sum of Flow (m^3^/s)	The Supply Time (d)
Pool 6	Hutuohe–Cihe	294.34	0.82	41.55
Pool 6–7	Hutuohe–Shahe (north)	466.53	0.99	54.54
Pool 6–8	Hutuohe–Tanghe	717.03	8.15	10.18
Pool 6–9	Hutuohe–Fangshuihe	600.11	8.15	12.08
Pool 6–10	Hutuohe–Puyanghe	891.44	10.94	12.08
Pool 6–11	Hutuohe–Gangtou Tunnel	1044.95	14.22	10.54

**Table 7:** Results of the second trial calculation.

Pool Number	The Name of Channel	The Sum of Volume (10^3^ Km^3^)	The Sum of Flow (m^3^/s)	The Supply Time (d)
Pool 9	Tanghe–Fangshuihe	133.58	0	-
Pool 9–10	Tanghe–Puyanghe	291.33	2.79	17.63
Pool 9–11	Tanghe–Gangtou Tunnel	578.42	2.82	11.03

**Table 8:** Local partition of water supply time.

Pool Number	The Name of Channel	The Sum of Volume (10^3^ Km^3^)	The Sum of Flow (m^3^/s)	The Supply Time (d)
Pool 6	Hutuohe–Cihe	294.34	0.82	41.55
Pool 7	Cihe–Shahe (north)	172.19	0.17	117.23
Pool 8	Shahe (north)–Tanghe	250.5	7.16	4.05
Pool 9–10	Tanghe–Puyanghe	424.91	2.79	17.63
Pool 11	Puyanghe–Gangtou Tunnel	153.51	3.28	5.42

## Data Availability

Not applicable.
